# Antibiotic prescribing appropriateness for urinary tract infections in a tertiary emergency department: a retrospective cohort study

**DOI:** 10.1186/s12873-026-01686-6

**Published:** 2026-07-25

**Authors:** Khulood Abdulghani, Maan Jamjoom, Shaza Dheya Alothmany, Razan Abdullah Filfilan, Abdullah Mohsen Alomairi, Bsaim Abdulsalam Altirkistani, Lama Hamoud Alsubhi, Faisal Fahad Aljadani, Jamil M. Baljoon, Sultan Alotaibi

**Affiliations:** 1 Department of Emergency Medicine, Ministry of the National Guard – Health Affairs, Jeddah, Saudi Arabia; 2 King Abdullah International Medical Research Center, Jeddah, Saudi Arabia; 3 Pharmaceutical Care Department, Ministry of the National Guard – Health Affairs, Jeddah, Saudi Arabia; 4 College of Medicine, King Saud bin Abdulaziz University for Health Sciences, Jeddah, Saudi Arabia; 5 Pediatric, Dalhousie University, Halifax, Nova Scotia Canada; 6 Emergency Medicine, King Fahad Armed Forces Hospital, Jeddah, Saudi Arabia

**Keywords:** Antimicrobial resistance, Urinary tract infections, Emergency department, Antibiotic prescription, Antimicrobial stewardship, Guideline adherence

## Abstract

**Introduction:**

Antibiotic misuse in emergency departments (EDs) drives antimicrobial resistance and adverse outcomes. This study assessed ED antibiotic prescribing adherence to hospital UTI guidelines at King Abdulaziz Medical City, Jeddah, Saudi Arabia, and identified predictors of non-adherence.

**Methods:**

This retrospective cohort study included consecutive patients undergoing urine culture in the ED between September and December 2021. Two independent clinical pharmacists assessed appropriateness using the validated National Antimicrobial Prescribing Survey (NAPS) tool against the Ministry of National Guard Health Affairs (MNGHA) 2022 UTI guidelines across four domains: drug selection, dose, route, and duration. Two binary logistic regression models identified independent predictors of non-adherence and 30-day ED revisit.

**Results:**

Of 346 patients (78.0% female), 184 (53.2%) had positive cultures and 216 (62.4%) received antibiotics. Overall, 153 (44.2%) had guideline-adherent management and 189 (54.6%) were non-adherent; among antibiotic-treated patients, only 31 (14.4%) received a guideline-adherent prescription. Leading non-adherence reasons were inappropriate drug selection (31.7%), overtreatment (29.6%), and resistance mismatch (13.8%). Positive culture (adjusted OR 1.89, *p* = 0.018) and UTI indication (adjusted OR 2.79, *p* < 0.001) independently predicted non-adherence. Positive-culture patients had higher 30-day ED revisit rates (26.6% vs. 14.8%, *p* = 0.008). Independent predictors of revisit were non-adherent prescribing (OR 2.20), pediatric age (OR 3.63), and prior antibiotic use (OR 2.91).

**Conclusions:**

Antibiotic prescribing for UTIs in this tertiary ED demonstrated significant non-adherence to MNGHA guidelines, attributable primarily to inappropriate drug selection and overtreatment of culture-negative patients, and was independently associated with 30-day ED revisit. Electronic decision support, pharmacist-led culture follow-up, and local antibiogram updates are needed to improve prescribing quality. Addressing these gaps is an actionable priority for antimicrobial stewardship in resource-limited ED settings and is directly relevant to curbing antimicrobial resistance and improving patient safety. As a single-centre retrospective study restricted to culture-ordered patients, findings may not generalise to all ED UTI presentations.

## Background

Timely administration of appropriate antibiotics in emergency departments (EDs) significantly affects patient outcomes. Antibiotics, the second most commonly prescribed medication in the EDs, accounted for 16% of all visits [1, 2]. However, more than one-third of these prescriptions were deemed inappropriate [3]. Antibiotic misuse, including inadequate dosing or incorrect duration, is prevalent, with up to 50% of the prescriptions in community and hospital settings considered unjustified [4]. In Saudi Arabia, a study in Riyadh found that 69.6% of antibiotic prescriptions were inaccurate [5, 6]. Even for self-limiting viral infections, antibiotics are frequently prescribed, as observed in a South African study [7]. Such inappropriate use can lead to adverse effects (AEs), including side effects, opportunistic infections, treatment failure, and antimicrobial resistance (AMR) [2, 6].

AMR, largely driven by irrational prescribing practices, is a growing global threat, projected to cause 9 million annual deaths worldwide by 2050 if left unaddressed [8]. Among uropathogens, AMR is of particular concern in Saudi Arabia [9, 10]. *Escherichia coli*, a leading cause of urinary tract infections (UTIs), exhibits resistance rates exceeding 30% for ciprofloxacin and 40% for trimethoprim-sulfamethoxazole in some regions. *Klebsiella pneumoniae* and *Enterococcus* species also show increasing multidrug resistance in hospital settings [7]. These resistance patterns complicate the empirical therapy and underscore the need for adherence to local treatment guidelines.

Several factors contribute to inappropriate prescriptions in EDs. For example, time pressures resulting in overprescription and inadequate diagnostic resources mask the underlying cause of bacterial infections, leading to the prescription of antibiotics as a preventive measure [9]. A U.S. study by Shehab et al. reported that over 50% of antibiotic prescriptions in ambulatory care were inappropriate or unnecessary, contributing to an estimated 142,505 annual ED visits for antibiotic-related AEs and 78.7% for allergic reactions [11]. Reducing unnecessary antibiotic use could slightly lower these risks [11].

Furthermore, emergency physicians, as frontline providers, often initiate empirical therapy using limited patient-specific data. Dumkow et al. highlighted their critical role in AMS but noted that optimal intervention targets in EDs remain unestablished [12]. ED stewardship requires unique strategies compared with inpatient settings [13]. Clinical pharmacist-led culture follow-up programs have demonstrated improved time-to-culture reviews, guideline-concordant prescriptions, and reduced return visits within 96 h [14].

While international studies consistently document guideline non-adherence rates of 30–70% for UTI prescribing in EDs, locally validated data from Saudi Arabian tertiary EDs remain limited. Given that regional uropathogens, particularly E. coli and K. pneumoniae, exhibit ciprofloxacin resistance rates exceeding 30% in some centers [13], site-specific evaluation of prescribing practices is necessary to inform locally relevant antimicrobial stewardship (AMS) strategies. Accordingly, this study aimed to assess the appropriateness of empiric antibiotic prescribing for UTI against MNGHA guidelines at King Abdulaziz Medical City, Jeddah.

## Methods

### Study design and setting

A retrospective cohort study design was employed to assess the appropriateness of antibiotic selection in patients with UTI presenting to the ED at King Abdulaziz Medical City in Jeddah. All consecutive patients meeting the inclusion criteria between September 1 and December 31, 2021, were included. The primary outcome was to assess the appropriateness of antibiotic prescriptions for UTI in the ED and to report whether the selected antibiotics were consistent with the Ministry of National Guard Health Affairs (MNGHA) guidelines for UTI and whether they were necessary. The MNGHA provides UTI management guidelines, recommending nitrofurantoin (100 mg twice daily for 5 days) or fosfomycin (3 g single dose) for uncomplicated UTIs in women, and ceftriaxone (1 g IV daily) or piperacillin-tazobactam (3.375 g IV every 6 h) for complicated UTIs. These recommendations are adjusted for local susceptibility and patient factors such as pregnancy or renal function. Treatment duration varies from 3 to 7 days for uncomplicated lower UTIs and 7–14 days for complicated UTIs or pyelonephritis, with follow-up cultures advised for persistent symptoms [15]. The guideline was developed by the infectious disease department of the MNGHA.

### Definition of antibiotic appropriateness and guideline adherence

Antibiotic appropriateness was operationalized using the validated National Antimicrobial Prescribing Survey (NAPS) tool, which evaluates prescriptions across four domains: (1) drug selection, (2) dose, (3) route of administration, and (4) treatment duration. A prescription was classified as Appropriate/Adherent if it matched MNGHA guideline recommendations for the patient’s clinical presentation category. Appropriately withholding antibiotics (e.g., for culture-negative cases or asymptomatic bacteriuria without pregnancy) was also classified as adherent. Urine culture ordering was classified as “necessary” when obtained for patients with documented urinary symptoms (e.g., dysuria, urgency, frequency, flank pain, fever) or recognized risk factors for complicated infection (e.g., pregnancy, indwelling catheterization, immunocompromised), and as “unnecessary” when ordered in the absence of such symptoms or risk factors, consistent with screening cultures not indicated by MNGHA criteria.

Non-adherence was categorized as: (a) wrong drug selection: an antibiotic not recommended for the identified or suspected pathogen/indication; (b) overtreatment: prescribing antibiotics for a culture-negative patient without clinical UTI symptoms, or use of unnecessarily broad-spectrum agents; (c) inadequate duration: treatment course shorter than guideline-recommended minimum; (d) inadequate disposition: outpatient management when guideline criteria indicated hospitalization; and (e) resistance mismatch: empiric antibiotic to which the isolated organism was found resistant on susceptibility testing.

Each case was independently assessed by two clinical pharmacists with subspecialty experience in emergency medicine and infectious diseases, using the standardized NAPS tool with predefined criteria for all four domains. Patient-level factors including pregnancy status, renal function, and relevant comorbidities were considered when these were specified within the MNGHA guideline recommendations. In cases of disagreement between the two assessors, consensus was reached through structured discussion; unresolved discrepancies were adjudicated by a third senior clinical pharmacist. A formal inter-rater reliability coefficient (kappa statistic) was not prospectively calculated, which is acknowledged as a methodological limitation; however, the use of the validated, structured NAPS tool with explicit operational criteria minimized subjectivity in classification and partially mitigated this limitation.

### Ethical approval and participant inclusion

Ethical approval was granted by the Institutional Review Board of King Abdullah International Medical Research Centre (KAIMRC), Jeddah, Saudi Arabia (IRB number NRJ23J/194/07). The requirement for informed patient consent was waived as this was a retrospective study using de-identified data retrieved from the hospital’s BestCare system. The inclusion criteria included patients who presented to the ED and underwent urine culture for presumed diagnosis of UTI between September 1, 2021, and December 31, 2021. Patients who were admitted to inpatient beds during this period were excluded. A consecutive sampling technique was utilized. A formal a priori sample size calculation was not performed, as this was a retrospective quality-improvement audit of practice rather than a hypothesis-driven study; the consecutive sampling approach instead aimed to capture the entire eligible cohort within a defined four-month audit period, maximizing representativeness within that window. All patients who met the criteria and duration were included.

### Data collection

The data required was gathered using the Electronic Medical Records System of the King Abdulaziz Medical City Bestcare System. The information was thereafter compiled into a spreadsheet for further analysis. Moreover, age group, gender, Charlson Comorbidities Index, antibiotic use within the last 30 days, name of antibiotic prescribed, susceptibility of antibiotic to culture, antibiotic appropriateness, and ED follow-up after antibiotic administration were the determined categorical variables. All the data was taken from the collection form and entered into a Microsoft Excel file for data accumulation. Data extraction was performed from the electronic medical record system by trained investigators and verified by a second reviewer. Records with incomplete antibiotic prescription details were reviewed manually.

### Statistical analysis

Descriptive statistics were calculated for all relevant variables and presented as mean ± standard deviation (SD) for normally distributed continuous variables, median (interquartile range) for non-normally distributed continuous variables, and frequency with percentage for categorical variables. Normality of continuous variables was assessed using the Shapiro-Wilk test. Categorical variables were compared between groups using Pearson’s chi-square test; Fisher’s exact test was applied when any expected cell count was below five. Continuous variables were compared using the independent samples t-test for normally distributed data or the Mann-Whitney U test for non-normal distributions. Two separate binary logistic regression models were constructed: the first to identify independent predictors of guideline non-adherence, and the second to identify independent predictors of 30-day ED revisit. Results are reported as adjusted odds ratios (OR) with 95% confidence intervals (CI). Model fit was assessed using the Nagelkerke R² and Hosmer-Lemeshow goodness-of-fit test. Multicollinearity was evaluated using the variance inflation factor (VIF); values exceeding 10 were considered indicative of problematic collinearity. A two-tailed p-value of < 0.05 was considered statistically significant for all analyses. All analyses were conducted using R version 4.5.2. The non-adherence model included six predictor variables with 93 outcome events (events per variable [EPV] = 15.5), and the revisit model included six predictors with 73 outcome events (EPV = 12.2), both exceeding the recommended minimum EPV of 10. The total cohort of 346 patients also provided sufficient power (> 90%) to detect moderate effect sizes (OR ≥ 1.5) in the primary chi-square comparisons at α = 0.05. Missing values were handled using case-wise exclusion for analyses requiring those variables.

## Results

### Demographics

This retrospective cohort study included 346 patients, of whom 270 (78.0%) were female. Full age, comorbidity, and antibiotic-exposure distributions are summarised in Table 1. Overall, 216 patients (62.4%) were treated with antibiotics and 130 (37.6%) were not.

**Table 1:** Study participants’ demographics (*N* = 346)

Characteristic	*N* (Percentage) / Mean ± SD
**Age Group (Years)**	
Adult (15–64)	200 (57.8%)
Elderly (> 64)	114 (33%)
Pediatric (< 15)	31 (9.0%)
**Gender**	
Female	270 (78%)
Male	76 (22%)
**Charlson Comorbidity Index Category**	
Low (0–1)	161 (47%)
Moderate (2–3)	60 (17%)
High (4+)	125 (36%)
Charlson Comorbidity Index (Mean ± SD)	2.48 ± 2.62
Prior Antibiotic Use (Within 30 Days)	65 (19%)
**Urine Culture Result**	
Negative	162 (47%)
Positive	184 (53%)
**Urine Culture Ordering**	
Unnecessary	143 (41%)
Necessary	203 (59%)
**Antibiotic Treatment**	
Antibiotic-treated patients	216 (62.4%)
No antibiotics treated	130 (37.6%)

### Antibiotic prescriptions in the ED

**Fig. 1:**
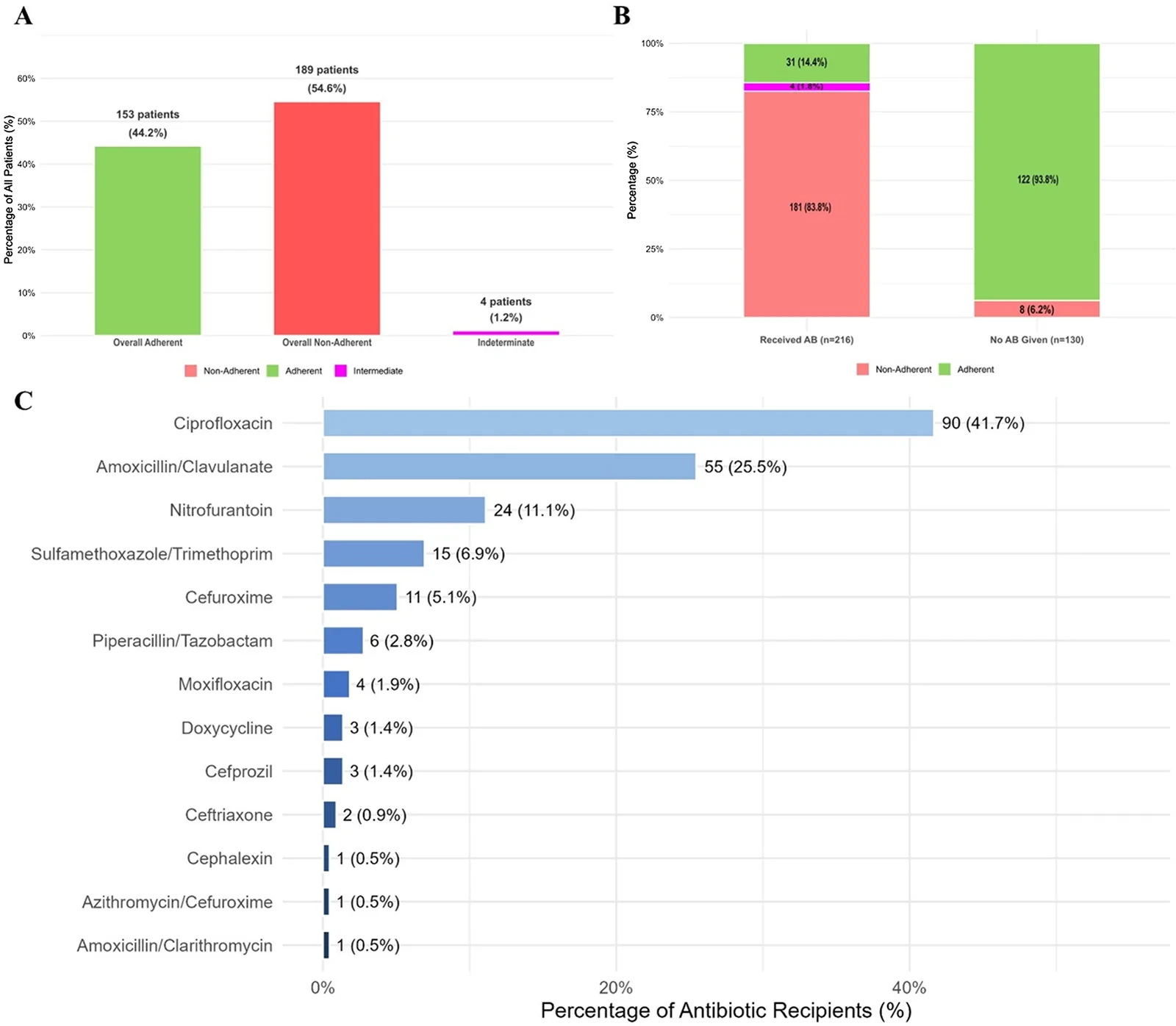
Antibiotic prescribing patterns and guideline adherence among 346 ED patients with urine cultures. Distribution of all 346 patients by **(A)** adherence and **(B)** antibiotic prescribing category. **(C)** Frequency of empiric antibiotics prescribed among the 216 patients who received an antibiotic in the ED

Of 346 patients, 153 (44.2%) had guideline-adherent overall management and 189 (54.6%) were non-adherent (Fig. 1A). Among the 216 antibiotic-treated patients, 31 (14.4%) received a guideline-adherent prescription and 181 (83.8%) received a non-adherent prescription; four cases (1.8%) were indeterminate (Fig. 1B). Among the 130 patients who received no antibiotic, 122 (93.8%) had this appropriately withheld and 8 (6.2%) should have received an antibiotic but did not. Of 162 culture-negative patients, 96 (59.3%) appropriately received no antibiotics. The most commonly prescribed agents were ciprofloxacin (90/216, 41.7%), amoxicillin-clavulanate (55/216, 25.5%), and nitrofurantoin (24/216, 11.1%) (Fig. 1C).

### Urine culture results and organism distribution

**Fig. 2:**
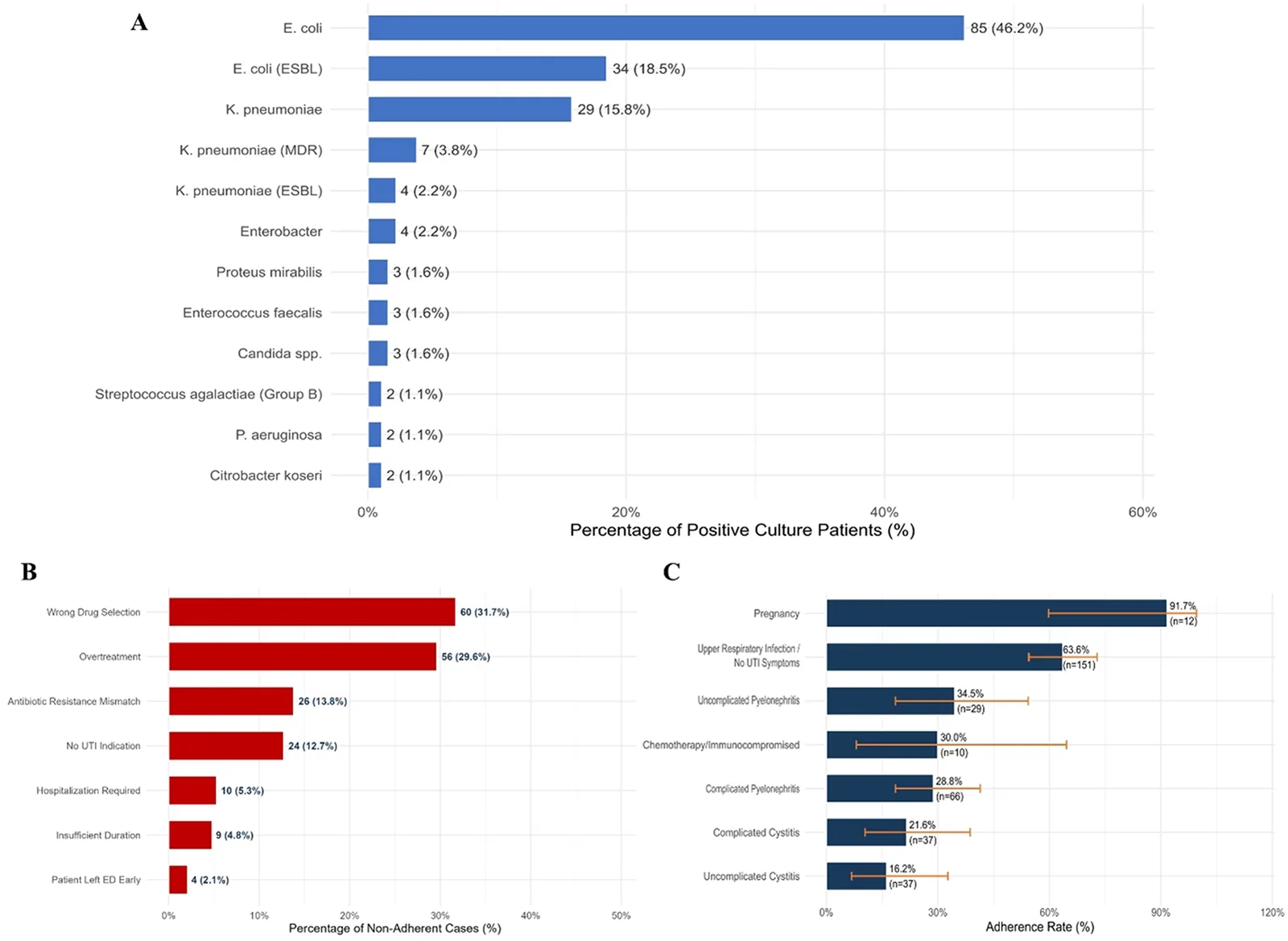
Microbiological profile of positive urine cultures and reasons for antibiotic non-adherence.** (A)** Distribution of organisms isolated from 184 patients with positive urine cultures. **(B)** Reasons for antibiotic non-adherence among 189 non-adherent cases. **(C)** Guideline adherence rates by clinical indication with 95% confidence intervals

Urine cultures were positive in 184 patients (53.2%) and negative in 162 (46.8%). Among positive cultures, the most frequently isolated organisms were *E. coli* (85/184, 46.2%), *E. coli* ESBL-producing (34/184, 18.5%), and *K. pneumoniae* (29/184, 15.8%). MDR organisms accounted for 7 positive cultures (3.8%). Of 147 patients with documented antibiotic susceptibility results, 45 (30.6%) received an antibiotic to which the isolated organism was resistant. Organism distribution is shown in Fig. 2A.

### Adherence to antibiotic guidelines

Of 162 culture-negative patients, 96 (59.3%) appropriately received no antibiotics. The 189 non-adherent cases comprise: 181 antibiotic-treated patients who received a guideline-discordant prescription and 8 patients who did not receive an antibiotic but should have (inadequate disposition or premature ED departure). Non-adherence was identified in 181 of 216 antibiotic-treated patients (83.8%), and a further 66 culture-negative patients (40.7%) received antibiotics without indication. The primary reasons for non-adherence were inappropriate drug selection (60 cases, 31.7%), overtreatment of culture-negative patients (56 cases, 29.6%), antibiotic resistance mismatch (26 cases, 13.8%), no UTI indication (24 cases, 12.7%), hospitalization required but not arranged (10 cases, 5.3%), insufficient treatment duration (9 cases, 4.8%), and patient leaving the ED early (4 cases, 2.1%). Non-adherence reasons are presented in Fig. 2B, with the lowest rates among patients with uncomplicated cystitis (6/37, 16.2%), complicated UTI (27/103, 26.2%), and uncomplicated pyelonephritis (10/29, 34.5%). Patients presenting without UTI symptoms or with upper respiratory infection (combined *n* = 151) demonstrated an adherence rate of 63.6%, largely driven by appropriate antibiotic withholding. Pregnant patients demonstrated the highest adherence of all groups (11/12, 91.7%), reflecting heightened prescriber awareness of fetal risk (Fig. 2C).

### Comparison of antibiotic usage between positive and negative urine cultures

Prior antibiotic use within 30 days was significantly more common among patients with positive cultures (54/184, 29.3%) compared to those with negative cultures (11/162, 6.8%; *p* < 0.0001). Guideline adherence differed markedly between groups: 57 of 184 positive-culture patients (31.0%) received guideline-concordant prescriptions, compared with 96 of 162 negative-culture patients (59.3%; *p* < 0.0001). These comparisons are summarized in Table 2.

**Table 2:** Patient characteristics and clinical outcomes stratified by urine culture result (*N* = 346)

Characteristic	Overall	Negative	Positive	*p*-value
	*N* = 346	*N* = 162	*N* = 184	
**Age Group (Years)**				
Adult (15–64)	200 (57.8%)	105 (65%)	95 (52%)	0.01
Elderly (> 64)	114 (33%)	40 (25%)	74 (40%)	
Pediatric (< 15)	31 (9.0%)	16 (9.9%)	15 (8.2%)	
Unknown	1	1	0	
**Gender**				
Female	270 (78%)	125 (77%)	145 (79%)	0.8
Male	76 (22%)	37 (23%)	39 (21%)	
**Charlson Comorbidity Index Category**				
Low (0–1)	161 (47%)	90 (56%)	71 (39%)	0.005
Moderate (2–3)	60 (17%)	26 (16%)	34 (18%)	
High (4+)	125 (36%)	46 (28%)	79 (43%)	
**Charlson Comorbidity Index (Mean ± SD)**	2.48 ± 2.62	2.01 ± 2.52	2.90 ± 2.64	0.001
**Prior Antibiotic Use**	65 (19%)	11 (7.0%)	54 (29%)	< 0.001
**Urine Culture Ordering**				
Unnecessary	143 (41%)	98 (60%)	45 (24%)	< 0.001
Necessary	203 (59%)	64 (40%)	139 (76%)	
**Adherence**				
Adherent	153 (44%)	96 (59%)	57 (31%)	< 0.001
Non-Adherent	189 (55%)	66 (41%)	123 (68%)	
Unknown	4	0	4	
**ED Revisit**	73 (21%)	24 (15%)	49 (27%)	0.011

Pearson’s Chi-squared test; t-test

### Follow up in the ED

**Fig. 3:**
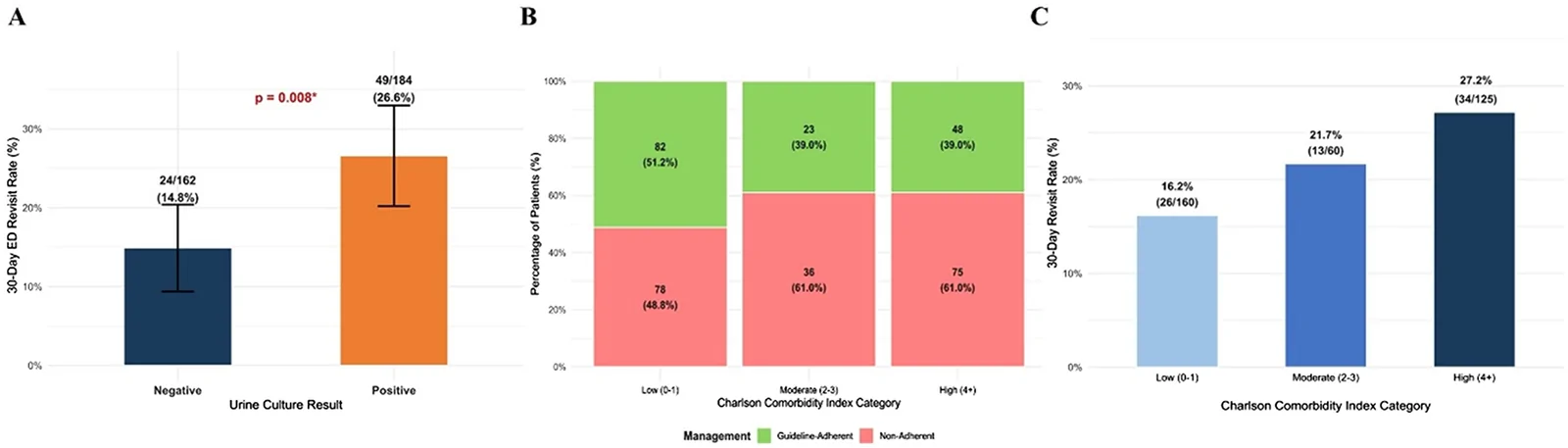
Clinical outcomes and comorbidity burden. **(A)** Thirty-day ED revisit rates stratified by urine culture result. **(B)** Guideline adherence stratified by Charlson Comorbidity Index (CCI) category. **(C)** Thirty-day ED revisit rates stratified by CCI category

Within 30 days of discharge, 73 patients (21.1%) returned to the ED. Positive-culture patients had a significantly higher revisit rate than negative-culture patients (49/184, 26.6% vs. 24/162, 14.8%; *p* = 0.008) (Fig. 3A).

### Comorbidity burden and outcomes

Non-adherence rates increased with comorbidity burden: 78/160 (48.8%) in low-CCI patients, 36/59 (61.0%) in moderate-CCI, and 75/123 (61.0%) in high-CCI, although this trend did not reach statistical significance (χ²=5.16, *p* = 0.076) (Fig. 3B). Similarly, the 30-day ED revisit rate increased with comorbidity burden: 26/160 (16.2%) in low-CCI, 13/60 (21.7%) in moderate-CCI, and 34/125 (27.2%) in high-CCI patients (χ²=5.06, *p* = 0.080). Subgroup denominators differ from the full cohort (*N* = 346) due to exclusion of 4 cases with indeterminate adherence status and 1 case with missing 30-day revisit data. These patterns are illustrated in Fig. 3C.

### Predictors of 30-day ED revisit

**Fig. 4:**
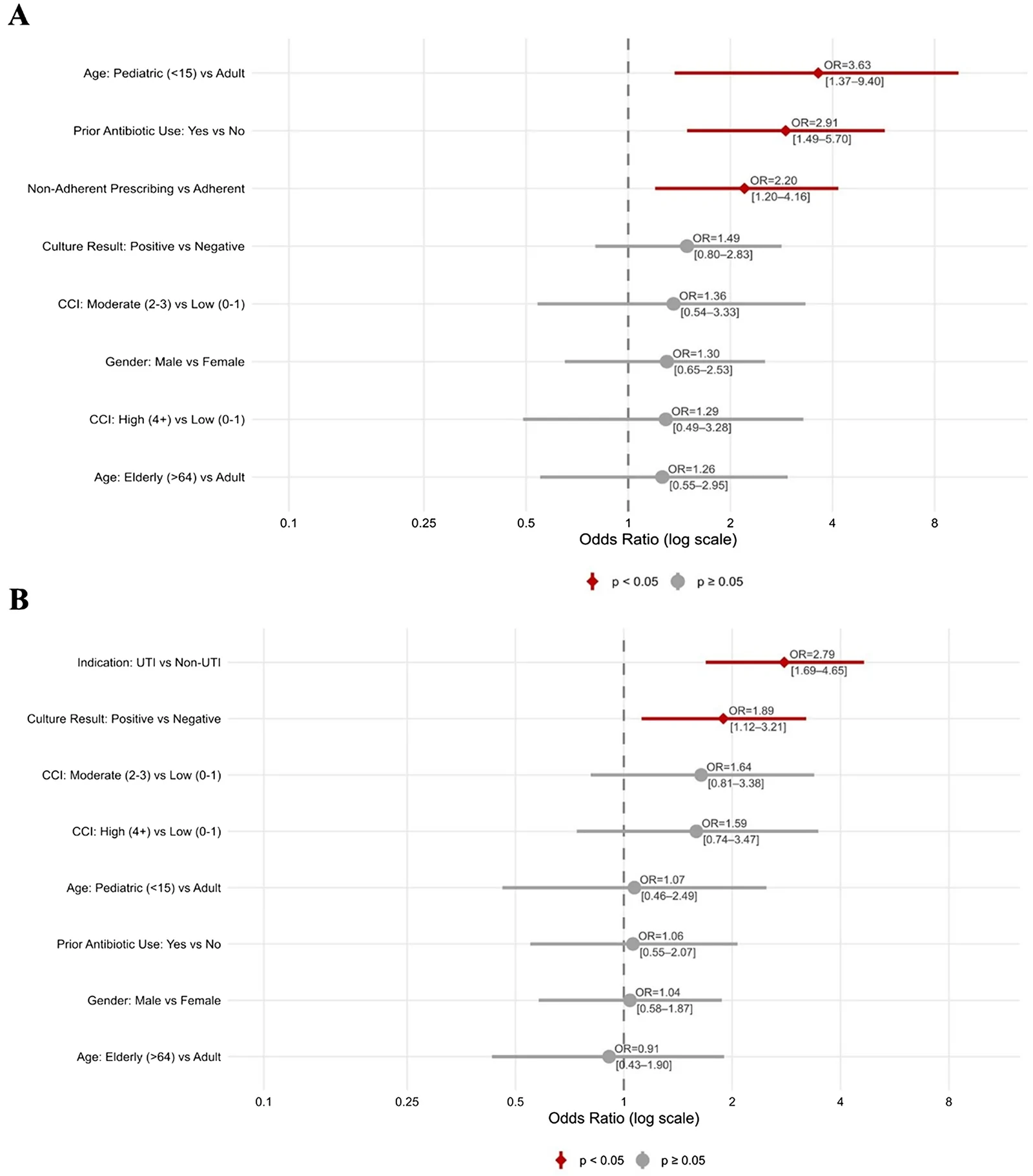
Forest plots of multivariable logistic regression results.** (A)** Adjusted odds ratios (OR) with 95% confidence intervals (CI) for independent predictors of 30-day ED revisit. **(B)** Adjusted odds ratios (OR) with 95% confidence intervals (CI) for independent predictors of guideline non-adherence. All models were adjusted for age group, gender, Charlson Comorbidity Index category, culture result, prior antibiotic use, and UTI indication. Values greater than 1.0 indicate increased odds of the outcome. Horizontal lines represent 95% CIs; the vertical dashed line at OR = 1.0 denotes the null effect. Red diamond represents a statistically significant association (*p* < 0.05), and grey circle represents a nonsignificant association

On multivariable logistic regression for 30-day ED revisit (Table 3), three variables independently predicted revisit: non-adherent prescribing (adjusted OR 2.20, 95% CI 1.20–4.16, *p* = 0.012), prior antibiotic use in the preceding 30 days (adjusted OR 2.91, 95% CI 1.49–5.70, *p* = 0.002), and pediatric age group (adjusted OR 3.63, 95% CI 1.37–9.40, *p* = 0.008). Elderly age group, gender, CCI category, and positive culture result were not independently associated with 30-day ED revisit after adjustment (Fig. 4A).

**Table 3:** Logistic regression revisit predictors

Predictor	Adjusted Odds Ratio	95% CI	*P* Value
Non-Adherent Prescribing	2.2	1.2–4.16	0.012
Elderly Age Group (> 64)	1.26	0.55–2.95	0.589
Pediatric Age Group (< 15)	3.63	1.37–9.4	0.008
Gender (Male)	1.3	0.65–2.53	0.444
CCI Category: Moderate (2–3)	1.36	0.54–3.33	0.498
CCI Category: High (4+)	1.29	0.49–3.28	0.6
Culture Result (Positive)	1.49	0.8–2.83	0.215
Prior Antibiotic Use in 30 days (Yes)	2.91	1.49–5.7	0.002

### Predictors of guideline non-adherence

On multivariable logistic regression for guideline non-adherence (Table 4), two variables independently predicted guideline non-adherence: positive urine culture result (adjusted OR 1.89, 95% CI 1.12–3.21, *p* = 0.018) and UTI clinical indication (adjusted OR 2.79, 95% CI 1.69–4.65, *p* < 0.001). Age group, gender, CCI category, and prior antibiotic use were not independently associated with non-adherence after adjustment (Fig. 4B).

**Table 4:** Logistic regression non-adherence predictors

Predictor	Adjusted Odds Ratio	95% CI	*P* Value
Age Group: Elderly (> 64)	0.91	0.43–1.9	0.8
Age Group: Pediatric (< 15)	1.07	0.46–2.49	0.876
Gender (Male)	1.04	0.58–1.87	0.892
CCI Category: Moderate (2–3)	1.64	0.81–3.38	0.175
CCI Category: High (4+)	1.59	0.74–3.47	0.241
Culture Result (Positive)	1.89	1.12–3.21	0.018
Prior Antibiotic Use in 30days (Yes)	1.06	0.55–2.07	0.854
UTI vs. NonUTI	2.79	1.69–4.65	< 0.001

## Discussion

This study identifies guideline non-adherence as a clinically consequential and modifiable target in antibiotic prescribing for UTIs in this tertiary ED. Of 346 patients, 189 (54.6%) received guideline-discordant care, and only 31 of 216 antibiotic-treated patients (14.4%) received a fully adherent prescription. The principal finding of this study is that non-adherent prescribing was independently associated with 30-day ED revisit (adjusted OR 2.20, 95% CI 1.20–4.16), alongside paediatric age and prior antibiotic use, positioning prescribing quality as a measurable and potentially modifiable lever for reducing downstream ED utilization.

Several interacting factors likely explain the high non-adherence rate. Empiric therapy was necessarily initiated before culture results were available (typically 24–72 h post-collection), requiring prescribers to rely on clinical presentation and prior probability of infection; this diagnostic uncertainty, combined with the high patient volumes and limited continuity of follow-up characteristic of ED practice, may reduce guideline consultation at the point of prescribing [11, 13, 16, 17]. The predominance of complicated presentations in our culture-ordered cohort (complicated UTI: 103/346, 29.8%; pyelonephritis: 29/346, 8.4%) likely compounded this uncertainty, as both indications carried non-adherence rates above the cohort average (26.2% and 34.5%, respectively; Fig. 2C). These findings align with international literature reporting UTI prescribing non-adherence of 30–70% in ED settings [1, 3].

Ciprofloxacin was the most frequently prescribed agent in our cohort (41.7%), despite its demotion to second-line status under the MNGHA 2022 guidelines and despite institutional susceptibility data showing that 30.6% of cultured organisms (45/147) were resistant to the empirically prescribed antibiotic, consistent with regional reports of *E. coli* ciprofloxacin resistance exceeding 30% [13]. ESBL-producing *E. coli* accounted for 18.5% of positive cultures and MDR organisms for a further 3.8%. As culture susceptibility data were unavailable at the time of prescribing, this pattern likely reflects entrenched pre-2022 prescribing habits and ciprofloxacin’s practical appeal (oral bioavailability, broad spectrum, twice-daily dosing) in time-pressured settings, consistent with prescribing patterns reported elsewhere [3, 12], rather than deliberate guideline deviation. Integrating real-time local antibiogram data into ED ordering systems is therefore a high-priority stewardship target [14].

Multivariable analysis identified positive urine culture result (adjusted OR 1.89, 95% CI 1.12–3.21, *p* = 0.018) and confirmed UTI indication (adjusted OR 2.79, 95% CI 1.69–4.65, *p* < 0.001) as independent predictors of non-adherence, while age, gender, CCI, and prior antibiotic use were not, suggesting that prescriber- and system-level factors may outweigh patient characteristics in driving non-adherence. This pattern was mirrored in the indication-level analysis: adherence ranged from 16.2% in cystitis to 91.7% in pregnancy (*p* < 0.001), with the high adherence in pregnancy plausibly reflecting heightened prescriber caution around teratogenic risk [16]. The comparatively low adherence in cystitis and complicated UTI (26.2%)—both indications with clear first-line MNGHA recommendations—suggests that targeted, indication-specific prescriber education may be a more effective stewardship lever than generic guideline dissemination.

The clinical relevance of non-adherence was most apparent in the 30-day ED revisit data: revisit rates were higher among non-adherently treated patients than adherently treated patients (27.5% vs. 13.7%; *p* = 0.003), and this association persisted after multivariable adjustment (adjusted OR 2.20, *p* = 0.012), alongside paediatric age (adjusted OR 3.63) and prior antibiotic use (adjusted OR 2.91). Revisit rates were also higher among positive-culture patients (26.6% vs. 14.8%; *p* = 0.008), consistent with the greater prescribing discordance observed in this subgroup (31.0% vs. 59.3% adherence in negative-culture patients). Reasons for revisit were not systematically documented and could not be retrospectively verified as related to the index UTI episode; these associations should therefore not be interpreted as evidence of a direct causal pathway between non-adherent prescribing and revisit, as they may partly reflect underlying illness severity or unrelated conditions. Prospective studies systematically capturing revisit aetiology are needed to clarify this relationship.

A clinically notable, though not statistically significant, gradient was also observed across comorbidity burden, with non-adherence rising from 48.8% in low-CCI patients to 61.0% in moderate- and high-CCI patients (*p* = 0.076), and revisit rates rising correspondingly from 16.2% to 27.2% (*p* = 0.080); larger, adequately powered studies are needed to confirm whether comorbidity burden independently amplifies these risks [18]. Notably, only 12 of 346 patients (3.5%) received pharmacist input, well below the approximately 22% intervention rates reported in comparable pharmacist-led culture follow-up programs achieving over 96% resolution [19, 20]. This gap, combined with the absence of a structured post-discharge culture review mechanism at our institution, represents a readily addressable stewardship target.

Based on these findings, priority stewardship interventions for this and similar ED settings include: (1) electronic clinical decision support integrated into the ED ordering system, providing real-time guideline-concordant suggestions informed by local resistance data; (2) pharmacist-led culture follow-up to address the 30.6% resistance mismatch rate and reduce preventable revisits; (3) regular institution-specific antibiogram updates, particularly for *E. coli* and ESBL-producing organisms; and (4) targeted prescriber education addressing the two dominant drivers of non-adherence—inappropriate drug selection (31.7%) and overtreatment of culture-negative patients (29.6%).

### Limitations and recommendations

This study has a few limitations, also. First, its retrospective design depends on complete medical records, risking documentation bias, especially for indication and severity. Second, only patients with urine cultures ordered in the ED were included, likely excluding uncomplicated UTI cases treated without cultures, which is common for straightforward cystitis in women. This creates a bias toward more complicated cases, overrepresenting non-adherence and limiting generalizability; consequently, these findings may not be generalizable to all ED UTI presentations, particularly uncomplicated cases managed empirically without culture confirmation. Third, antibiotic allergy data were missing, possibly explaining some non-adherence cases, like using second-line agents in penicillin-allergic patients. Fourth, symptom severity and clinical scores weren’t systematically collected, preventing analysis by clinical acuity. Fifth, provider details such as speciality, training, and rationale were not recorded, limiting understanding of prescriber behavior. Sixth, although the two assessing pharmacists used a standardized, structured tool with predefined criteria, a formal inter-rater reliability coefficient (kappa statistic) was not prospectively calculated. To address these issues and the high non-adherence rate (54.6%), we recommend ED-specific antimicrobial stewardship (AMS) strategies, including electronic decision support, pharmacist review, ongoing physician training, and local antibiograms [21]. These strategies are crucial given high antibiotic resistance, aiming to improve adherence, reduce revisits, and combat resistance. Future studies should assess these interventions’ impact, and patient factors like comorbidities and previous antibiotic use should be included to improve outcomes and reduce unnecessary antibiotics and adverse events.

## Conclusion

In this tertiary ED cohort, antibiotic prescribing for UTIs demonstrated significant guideline non-adherence. The findings show that 54.6% of UTI-related prescribing decisions were guideline-discordant, attributable primarily to inappropriate drug selection (31.7%) and overtreatment of culture-negative patients (29.6%). Non-adherent prescribing was independently associated with 30-day ED revisit (adjusted OR 2.20, 95% CI 1.20–4.16), alongside pediatric age (adjusted OR 3.63) and prior antibiotic use (adjusted OR 2.91); given the retrospective design, this association should not be interpreted as evidence of a causal relationship. These findings support the implementation of electronic clinical decision support, pharmacist-led culture follow-up programs, and targeted prescriber education as priority antimicrobial stewardship interventions in this and similar tertiary ED settings.

## Data Availability

The datasets used and/or analyzed in the current study are available from the corresponding author upon reasonable request.

## Abbreviations

AE: Adverse events
AMR: Antimicrobial resistance
AMS: Antimicrobial stewardship
ED: Emergency department
KAIMRC: King Abdullah International Medical Research Centre
MNGHA: Ministry of National Guard Health Affairs
UTI: Urinary tract infection
